# Supplementary material for: Crystal structures and fragment screening of SARS-CoV-2 NSP14 reveal details of exoribonuclease activation and mRNA capping and provide starting points for antiviral drug development
Source: Nucleic Acids Res. 2022 Dec 22;51(1):475–87. doi: 10.1093/nar/gkac1207 (PMC9841433; doi:10.1093/nar/gkac1207)
Supplement: gkac1207_Supplemental_Files [file gkac1207_supplemental_files.zip › NSP14-SupplementaryInformation-review4.docx]

**Supplementary Information for: Crystal structures and Fragment screening of SARS-CoV-2 NSP14 reveals details of exoribonuclease activation and mRNA capping and provides starting points for antiviral drug development**


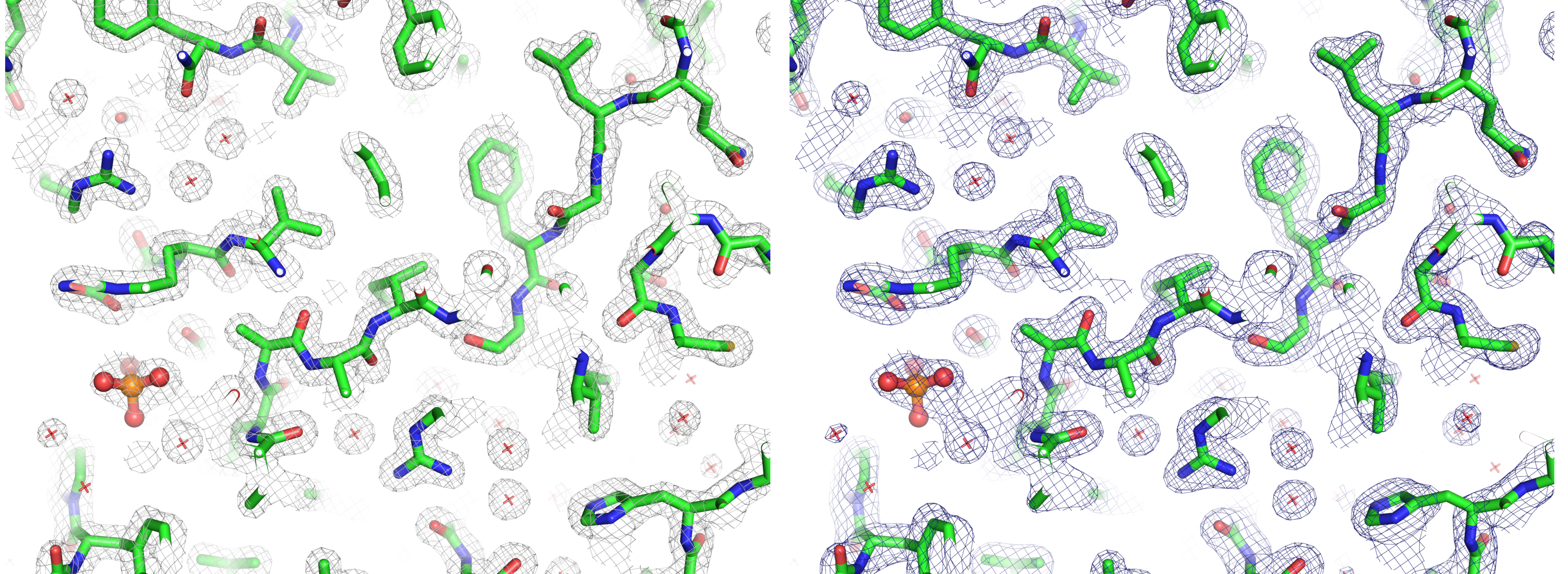


**Figure S1 –** Representative 2F_o_-1F_c_ electron density maps of NSP14 contoured at 1.5 σ. On the left hand panel is the final refined map in grey and the right hand panel shows a simulated annealing composite omit map in blue.


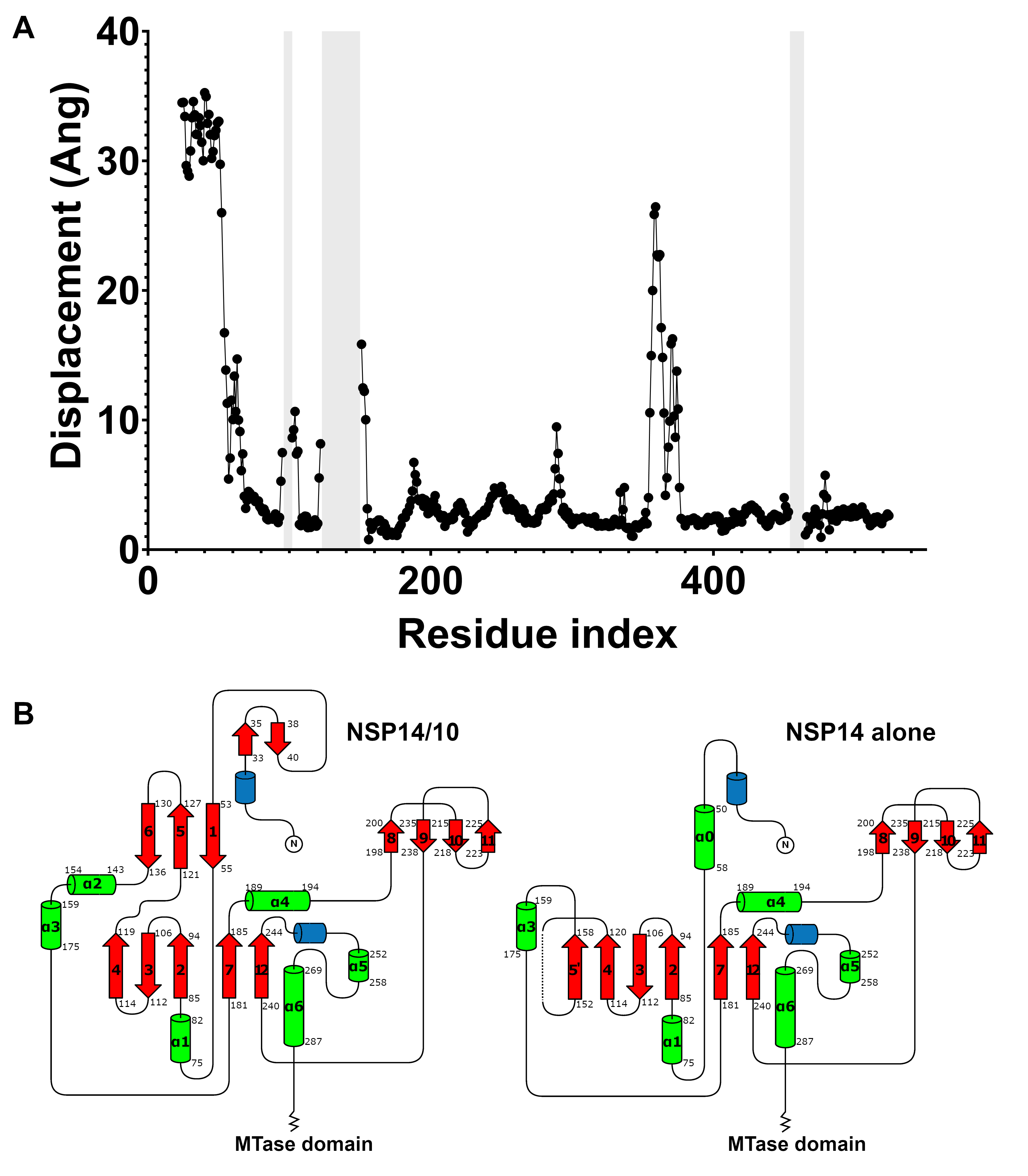


**Figure S2 -** Comparison of NSP14 alone with NSP14 in the NSP14/NSP10 complex. (**A**) Cα displacements in Angstrom plotted as a function of residue number. The shaded grey areas represent regions disordered in the NSP14 structure. (**B**) Topology diagram showing the changes in secondary structure elements between NSP14/NSP10 complex and NSP14 alone. For consistency the nomenclature of the complex has been applied to the NSP14 alone structure.


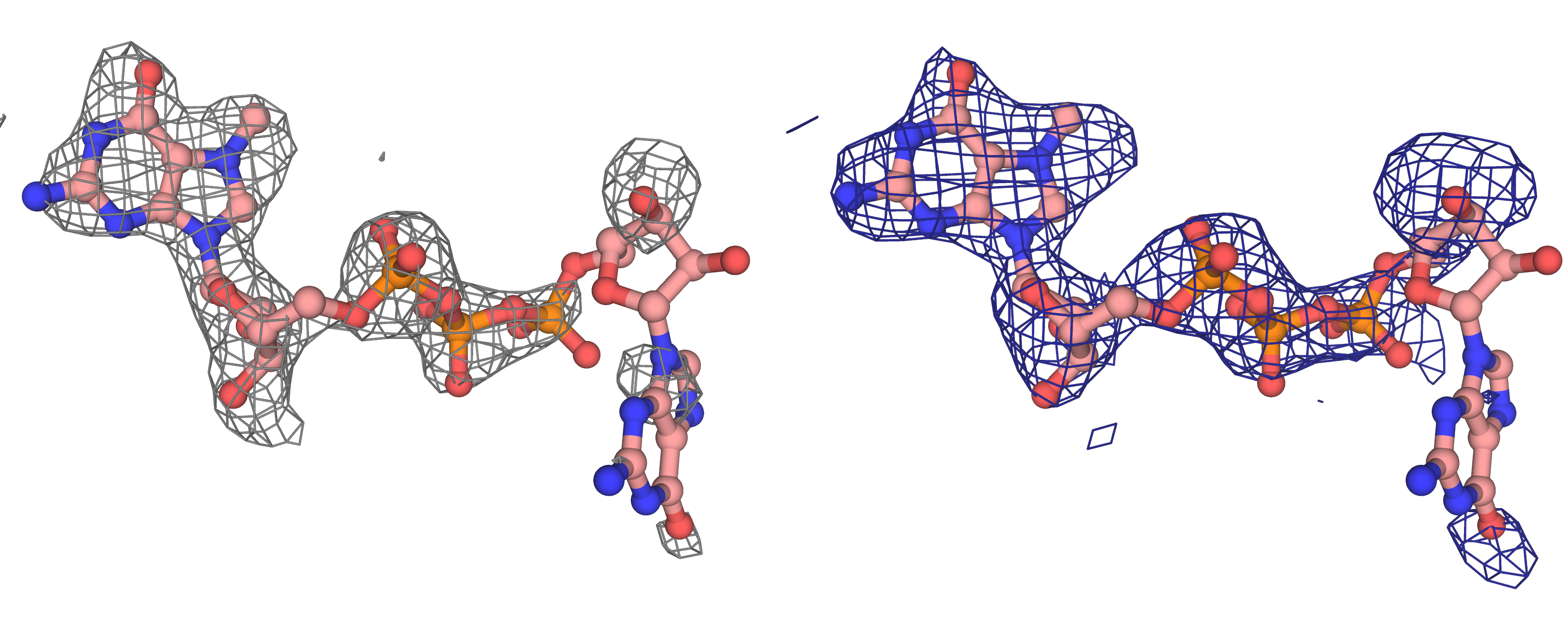


**Figure S3 –** 2F_o_-1F_c_ electron density maps at 2.5 Å resolution contoured at 1σ in the vicinity of the ^7Me^GpppG. The left hand panel shows the final refined map in grey and the right hand panel shows a simulated annealing composite omit map in blue. The methyl bulge is clearly visible in the electron density whilst the ribose and Guanine of the base 1 are less well ordered.


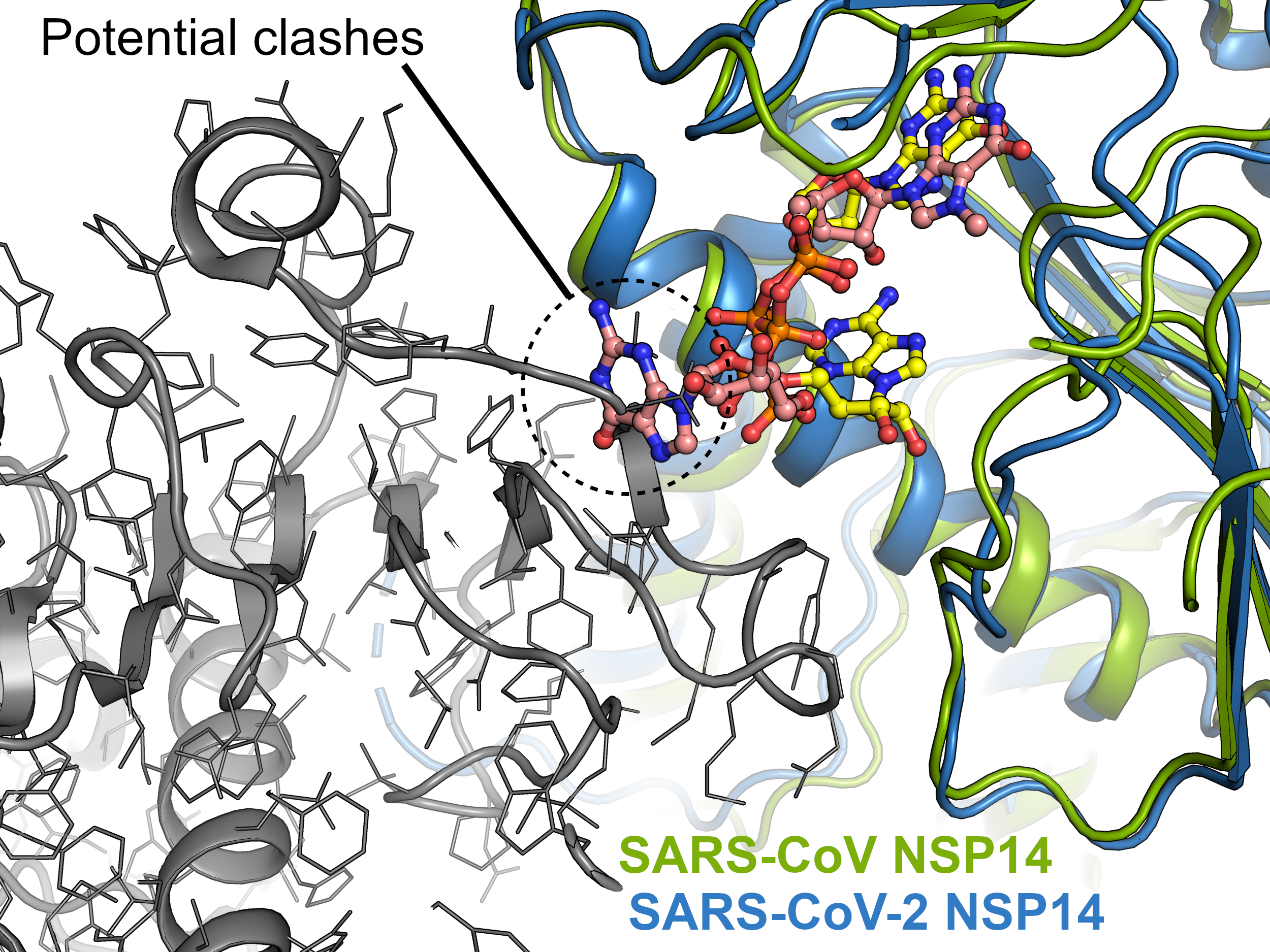


**Figure S4 -** Comparison of SARS-CoV-2 NSP14 (Blue with pink ^7Me^GpppG) with SARS-CoV NSP14 (Green with yellow GpppA). The symmetry mate of SARS-CoV NSP14 (shown in grey) approaches close to the free ribose O3’ and would appear to prevent the ^7Me^GpppG conformation being adopted in the SARS-CoV-2 NSP14 due to steric clashes.

| NSP14 DNA sequence | ATGCACCATCATCATCATCATTCTTCTGGTGTGGATAACAAGTTCAACAAGGAGCGTCGAAGAGCTCGCCGTGAAATTCGCCATCTGCCGAACCTGAACCGCGAACAGCGTCGCGCATTTATTCGCAGCCTGCGCGATGATCCGAGCCAGAGCGCGAACCTGCTGGCGGAAGCGAAGAAGCTGAACGATGCGCAGCCGAAGGGTACCGAGAACCTGTACTTCCAATCCATGCTGTTTAAAGATTGTAGTAAAGTCATTACCGGGCTGCATCCGACCCAAGCACCAACACATCTGAGTGTCGACACCAAGTTCAAAACGGAAGGGCTGTGCGTAGATATTCCCGGTATTCCAAAAGATATGACTTATCGCCGTCTGATTAGCATGATGGGCTTCAAAATGAATTACCAAGTGAACGGTTATCCGAATATGTTCATTACCCGCGAGGAAGCTATTCGCCATGTGCGCGCATGGATCGGCTTCGACGTGGAGGGATGTCATGCGACCCGCGAAGCCGTGGGGACTAACTTACCGCTGCAGCTGGGCTTTTCTACCGGCGTGAATTTAGTTGCCGTACCGACCGGATATGTAGACACGCCGAACAACACGGATTTTTCGCGTGTGTCAGCCAAACCACCTCCGGGCGATCAATTTAAGCACCTGATCCCACTGATGTACAAAGGCTTACCATGGAATGTTGTACGCATTAAGATCGTGCAGATGTTGAGCGATACTCTGAAAAATTTGTCGGATCGGGTAGTGTTTGTCCTGTGGGCGCACGGCTTCGAGCTTACTTCGATGAAATACTTTGTCAAGATCGGACCGGAACGTACCTGTTGTCTGTGCGACCGTCGCGCCACCTGTTTCAGTACCGCGTCTGACACTTATGCGTGTTGGCACCACTCGATTGGTTTTGATTACGTATACAATCCGTTTATGATCGATGTCCAACAATGGGGCTTTACGGGGAATTTGCAGTCTAATCACGATCTGTACTGTCAGGTACACGGCAATGCCCACGTCGCCAGCTGTGACGCTATTATGACCCGTTGCCTGGCTGTCCACGAATGTTTCGTGAAACGTGTTGATTGGACCATTGAATACCCTATTATCGGTGACGAGCTGAAAATTAACGCTGCGTGCCGTAAAGTTCAACACATGGTGGTCAAAGCGGCCCTGTTGGCCGATAAATTCCCAGTTTTGCACGACATTGGCAATCCTAAAGCAATCAAATGTGTTCCCCAAGCTGATGTGGAATGGAAATTCTACGATGCACAGCCATGCTCCGACAAGGCTTACAAAATTGAAGAACTGTTCTATAGCTATGCCACCCATTCTGACAAATTCACGGATGGTGTTTGTCTGTTTTGGAACTGTAACGTTGATCGTTATCCGGCAAACTCCATTGTGTGTCGCTTTGATACTCGGGTCCTCTCAAATCTCAATTTACCCGGCTGCGATGGCGGTTCTCTGTACGTGAATAAACACGCCTTCCATACTCCGGCATTTGATAAATCAGCGTTCGTCAATTTAAAGCAGCTGCCGTTTTTCTATTATAGCGATAGCCCCTGCGAAAGCCATGGGAAACAGGTGGTTAGCGATATCGACTATGTCCCGCTGAAGTCGGCCACGTGTATCACCCGTTGTAACTTAGGAGGCGCAGTTTGCCGTCACCATGCGAACGAGTACCGGCTGTATTTAGACGCATATAACATGATGATTTCTGCCGGCTTCAGTCTTTGGGTTTACAAACAGTTCGATACCTATAATCTGTGGAACACGTTTACTCGTCTCCAATAACAGTAAAGGTGGATACGGATCCGAATTCGAGCTCCGTCGACAAGCTTGCGGCCGCACTCGAGCACCACCACCACCACCACTGA |
| --- | --- |
| NSP14 Protein sequence | **MHHHHHHSSGVDNKFNKERRRARREIRHLPNLNREQRRAFIRSLRDDPSQSANLLAEAKKLNDAQPKGTENLYFQ**SMLFKDCSKVITGLHPTQAPTHLSVDTKFKTEGLCVDIPGIPKDMTYRRLISMMGFKMNYQVNGYPNMFITREEAIRHVRAWIGFDVEGCHATREAVGTNLPLQLGFSTGVNLVAVPTGYVDTPNNTDFSRVSAKPPPGDQFKHLIPLMYKGLPWNVVRIKIVQMLSDTLKNLSDRVVFVLWAHGFELTSMKYFVKIGPERTCCLCDRRATCFSTASDTYACWHHSIGFDYVYNPFMIDVQQWGFTGNLQSNHDLYCQVHGNAHVASCDAIMTRCLAVHECFVKRVDWTIEYPIIGDELKINAACRKVQHMVVKAALLADKFPVLHDIGNPKAIKCVPQADVEWKFYDAQPCSDKAYKIEELFYSYATHSDKFTDGVCLFWNCNVDRYPANSIVCRFDTRVLSNLNLPGCDGGSLYVNKHAFHTPAFDKSAFVNLKQLPFFYYSDSPCESHGKQVVSDIDYVPLKSATCITRCNLGGAVCRHHANEYRLYLDAYNMMISAGFSLWVYKQFDTYNLWNTFTRLQ |

| NSP10 DNA Sequence | ATGTCCCCGATCCTGGGTTACTGGAAAATCAAAGGTCTGGTCCAGCCGACGCGGCTGTTGCTTGAATACTTAGAGGAGAAATATGAGGAACATCTGTATGAACGTGATGAAGGCGACAAATGGCGTAATAAAAAATTCGAGTTAGGGTTAGAGTTCCCCAATTTGCCGTATTATATCGACGGGGACGTGAAATTGACTCAGAGCATGGCCATCATTCGGTACATCGCAGATAAGCATAATATGTTAGGTGGCTGCCCTAAAGAACGGGCAGAAATCTCTATGCTGGAAGGTGCCGTGCTCGATATTCGTTACGGGGTATCCCGCATTGCCTATAGTAAAGATTTTGAAACCCTGAAAGTAGACTTTCTGTCTAAACTCCCCGAAATGCTTAAAATGTTTGAGGATCGCCTGTGCCACAAAACTTATCTGAACGGGGATCACGTGACCCATCCGGATTTTATGCTCTATGATGCGTTAGACGTGGTGCTTTACATGGATCCGATGTGTTTAGATGCGTTTCCGAAACTGGTGTGCTTCAAAAAACGGATTGAAGCTATCCCACAAATCGACAAGTATTTAAAAAGCAGCAAATATATCGCCTGGCCCTTACAGGGCTGGCAAGCTACGTTCGGTGGCGGCGATCATCCACCGAAGTCGGATCTGGAGGTGCTGTTTCAAGGCCCGCTGCGTAGCGCGGGAAATGCCACCGAAGTCCCAGCAAATTCTACCGTACTGTCATTTTGTGCATTTGCCGTGGACGCGGCGAAGGCCTATAAGGATTATCTGGCATCGGGTGGTCAGCCGATTACCAATTGTGTCAAAATGCTGTGCACGCATACGGGCACAGGGCAAGCAATCACAGTCACGCCGGAAGCCAACATGGATCAGGAGTCTTTTGGTGGCGCGAGCTGCTGCCTGTACTGCCGTTGTCACATTGATCATCCGAACCCGAAAGGTTTCTGTGATTTGAAAGGTAAATACGTACAAATCCCAACGACCTGTGCCAACGATCCGGTGGGCTTTACCCTGAAAAACACGGTCTGCACTGTGTGTGGCATGTGGAAAGGTTACGGCTGCTCTTGTGATCAGCTGCGTGAACCGATGTTACAATGA |
| --- | --- |
| NSP10 Protein Sequence | **MSPILGYWKIKGLVQPTRLLLEYLEEKYEEHLYERDEGDKWRNKKFELGLEFPNLPYYIDGDVKLTQSMAIIRYIADKHNMLGGCPKERAEISMLEGAVLDIRYGVSRIAYSKDFETLKVDFLSKLPEMLKMFEDRLCHKTYLNGDHVTHPDFMLYDALDVVLYMDPMCLDAFPKLVCFKKRIEAIPQIDKYLKSSKYIAWPLQGWQATFGGGDHPPKSDLEVLFQ**GPLRSAGNATEVPANSTVLSFCAFAVDAAKAYKDYLASGGQPITNCVKMLCTHTGTGQAITVTPEANMDQESFGGASCCLYCRCHIDHPNPKGFCDLKGKYVQIPTTCANDPVGFTLKNTVCTVCGMWKGYGCSCDQLREPMLQ* |
| NSP14_bicistronic DNA Sequence | CATATGCACCATCATCATCATCATTCTTCTGGTGTAGATCTGGGTACCGAGAACCTGTACTTCCAATCCATGGCGGAAAATGTGACTGGTCTGTTTAAAGATTGTAGTAAAGTCATTACCGGGCTGCATCCGACCCAAGCACCAACACATCTGAGTGTCGACACCAAGTTCAAAACGGAAGGGCTGTGCGTAGATATTCCCGGTATTCCAAAAGATATGACTTATCGCCGTCTGATTAGCATGATGGGCTTCAAAATGAATTACCAAGTGAACGGTTATCCGAATATGTTCATTACCCGCGAGGAAGCTATTCGCCATGTGCGCGCATGGATCGGCTTCGACGTGGAGGGATGTCATGCGACCCGCGAAGCCGTGGGGACTAACTTACCGCTGCAGCTGGGCTTTTCTACCGGCGTGAATTTAGTTGCCGTACCGACCGGATATGTAGACACGCCGAACAACACGGATTTTTCGCGTGTGTCAGCCAAACCACCTCCGGGCGATCAATTTAAGCACCTGATCCCACTGATGTACAAAGGCTTACCATGGAATGTTGTACGCATTAAGATCGTGCAGATGTTGAGCGATACTCTGAAAAATTTGTCGGATCGGGTAGTGTTTGTCCTGTGGGCGCACGGCTTCGAGCTTACTTCGATGAAATACTTTGTCAAGATCGGACCGGAACGTACCTGTTGTCTGTGCGACCGTCGCGCCACCTGTTTCAGTACCGCGTCTGACACTTATGCGTGTTGGCACCACTCGATTGGTTTTGATTACGTATACAATCCGTTTATGATCGATGTCCAACAATGGGGCTTTACGGGGAATTTGCAGTCTAATCACGATCTGTACTGTCAGGTACACGGCAATGCCCACGTCGCCAGCTGTGACGCTATTATGACCCGTTGCCTGGCTGTCCACGAATGTTTCGTGAAACGTGTTGATTGGACCATTGAATACCCTATTATCGGTGACGAGCTGAAAATTAACGCTGCGTGCCGTAAAGTTCAACACATGGTGGTCAAAGCGGCCCTGTTGGCCGATAAATTCCCAGTTTTGCACGACATTGGCAATCCTAAAGCAATCAAATGTGTTCCCCAAGCTGATGTGGAATGGAAATTCTACGATGCACAGCCATGCTCCGACAAGGCTTACAAAATTGAAGAACTGTTCTATAGCTATGCCACCCATTCTGACAAATTCACGGATGGTGTTTGTCTGTTTTGGAACTGTAACGTTGATCGTTATCCGGCAAACTCCATTGTGTGTCGCTTTGATACTCGGGTCCTCTCAAATCTCAATTTACCCGGCTGCGATGGCGGTTCTCTGTACGTGAATAAACACGCCTTCCATACTCCGGCATTTGATAAATCAGCGTTCGTCAATTTAAAGCAGCTGCCGTTTTTCTATTATAGCGATAGCCCCTGCGAAAGCCATGGGAAACAGGTGGTTAGCGATATCGACTATGTCCCGCTGAAGTCGGCCACGTGTATCACCCGTTGTAACTTAGGAGGCGCAGTTTGCCGTCACCATGCGAACGAGTACCGGCTGTATTTAGACGCATATAACATGATGATTTCTGCCGGCTTCAGTCTTTGGGTTTACAAACAGTTCGATACCTATAATCTGTGGAACACGTTTACTCGTCTCCAATAATAATAATTTAAGGAGATATACATGGCGGGAAACGCGACCGAAGTGCCTGCAAATTCTACAGTGCTGTCCTTCTGTGCCTTTGCTGTTGACGCGGCGAAGGCATACAAAGACTATCTCGCGTCTGGAGGCCAGCCGATCACAAATTGCGTCAAAATGCTGTGTACACACACCGGCACGGGTCAGGCAATTACCGTAACCCCGGAAGCCAACATGGATCAGGAAAGTTTCGGCGGAGCAAGCTGTTGCCTCTATTGTCGTTGCCATATCGACCACCCCAACCCGAAAGGCTTTTGCGATTTAAAAGGCAAGTATGTGCAGATCCCGACGACCTGCGCGAACGATCCGGTGGGCTTCACGTTAAAAAACACCGTGTGCACCGTGTGTGGTATGTGGAAAGGCTATGGCTGTAGTTGTGATCAGTTACGCGAGCCAATGTTGCAGTAACAGTAAAGGTGGATACGGATCCGAA |
| NSP14_bicistronic Protein Sequence | **MHHHHHHSSGVDLGTENLYFQ**SMAENVTGLFKDCSKVITGLHPTQAPTHLSVDTKFKTEGLCVDIPGIPKDMTYRRLISMMGFKMNYQVNGYPNMFITREEAIRHVRAWIGFDVEGCHATREAVGTNLPLQLGFSTGVNLVAVPTGYVDTPNNTDFSRVSAKPPPGDQFKHLIPLMYKGLPWNVVRIKIVQMLSDTLKNLSDRVVFVLWAHGFELTSMKYFVKIGPERTCCLCDRRATCFSTASDTYACWHHSIGFDYVYNPFMIDVQQWGFTGNLQSNHDLYCQVHGNAHVASCDAIMTRCLAVHECFVKRVDWTIEYPIIGDELKINAACRKVQHMVVKAALLADKFPVLHDIGNPKAIKCVPQADVEWKFYDAQPCSDKAYKIEELFYSYATHSDKFTDGVCLFWNCNVDRYPANSIVCRFDTRVLSNLNLPGCDGGSLYVNKHAFHTPAFDKSAFVNLKQLPFFYYSDSPCESHGKQVVSDIDYVPLKSATCITRCNLGGAVCRHHANEYRLYLDAYNMMISAGFSLWVYKQFDTYNLWNTFTRLQ |

**Supplementary Table 1**: DNA and protein sequences for the codon optimized NSP14 construct used in this study. The bold sequence is removed during purification by TEV protease cleavage.

| **PDBID** | **Ligand** | **Pocket** | **2Fo-Fc map (0.7σ)** | **Location** |
| --- | --- | --- | --- | --- |
| 5KSW |   Z1272494722 | 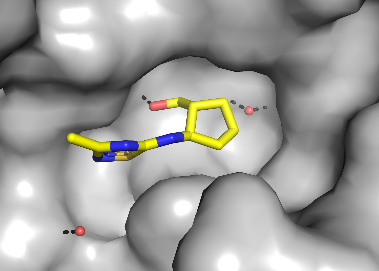 |   2.09 Å | 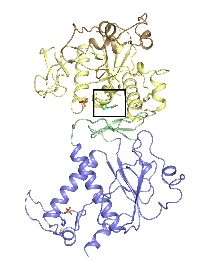  Hinge Pocket 1 |
| 5SKX |   Z126932614 | 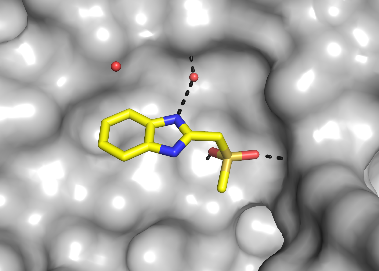 |   2.34 Å | 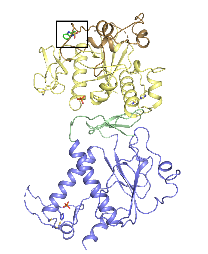  NSP10 Interface |
| 5SKY |   Z466628048 | 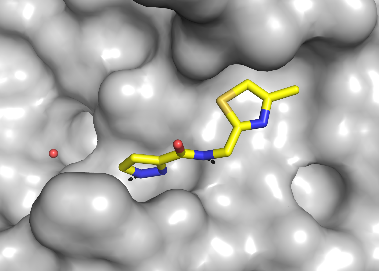 |   2.25 Å | 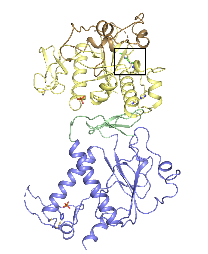  ExoN Active |
| 5SKZ |   Z57258487 | 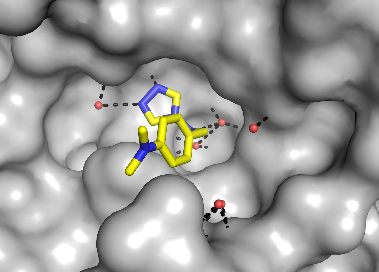 |   1.96 Å | 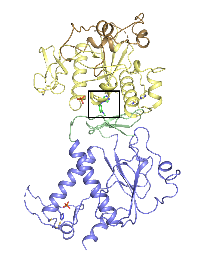  Hinge Pocket 1 |
| 5SL0 |   Z57260516 | 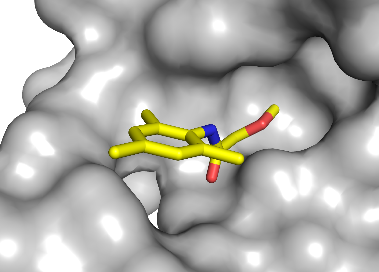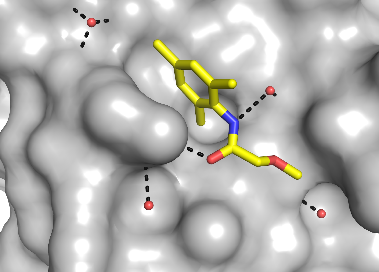 | 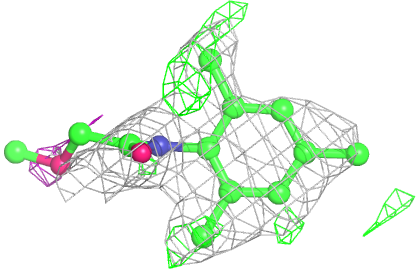  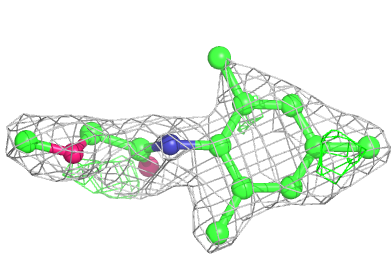  2.00 Å | 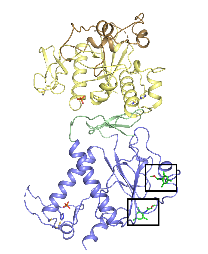  MTase Allosteric & MTase Other |
| 5SL1 |   Z1273312153 | 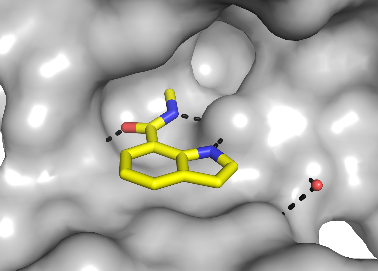 |   2.38 Å | 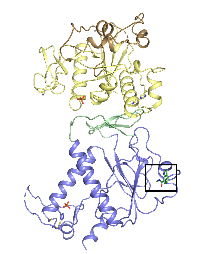  MTase Allosteric |
| 5SL2 |   Z100643660 | 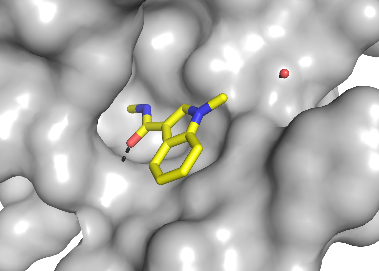 |   1.74 Å | 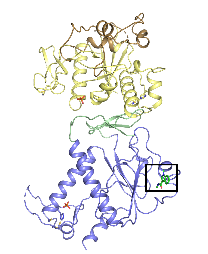 MTase Allosteric |
| 5SL3 |   Z223688272 | 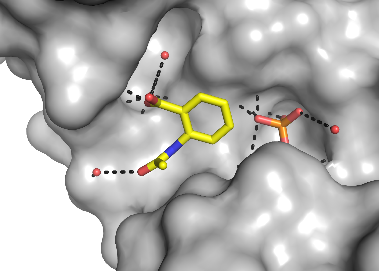 |   1.99 Å | 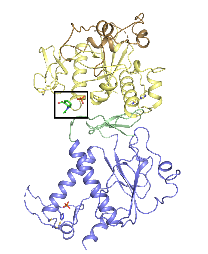  Hinge Pocket 2 |
| 5SL4 |   Z383202616 | 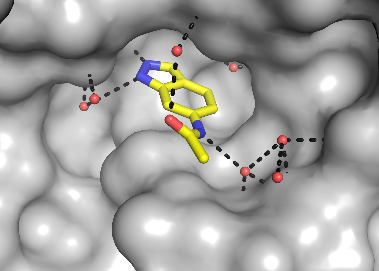  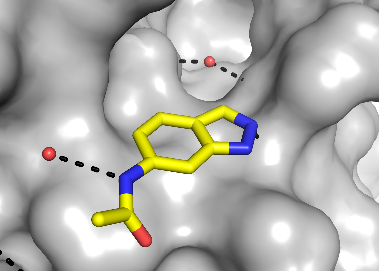 |     1.94 Å | 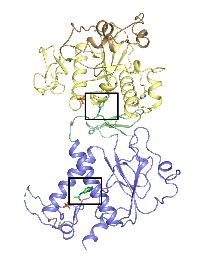  Hinge Pocket 1 & MTase Other |
| 5SL5 |   Z32014663 | 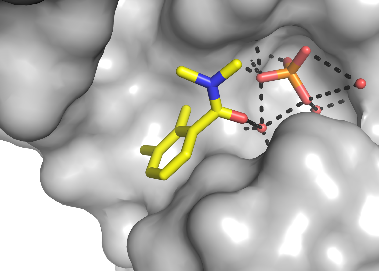 |   2.36 Å | 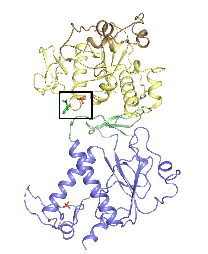  Hinge Pocket 2 |
| 5SL6 |   Z256709556 | 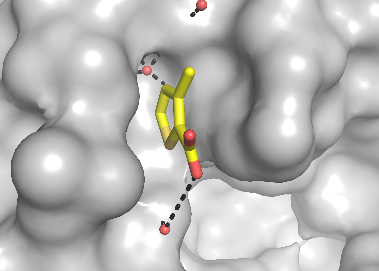  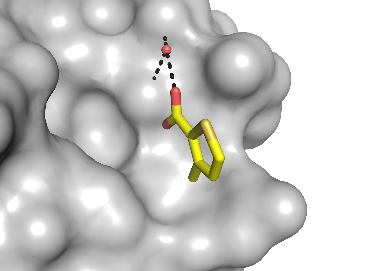 |     2.29 Å | 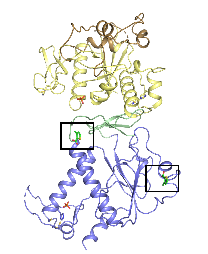  MTase Active & MTase Allosteric |
| 5SL7 |   Z1186029914 | 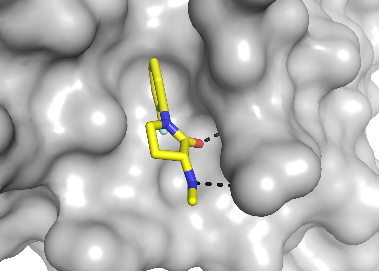 |   1.84 Å | 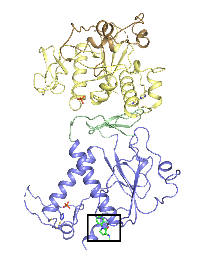  MTase Other |
| 5SL8 |   Z2856434762 | 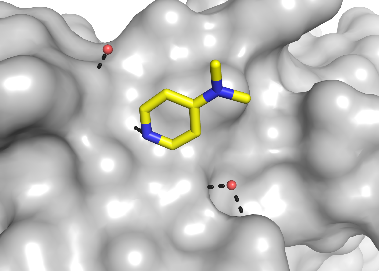 |   2.07 Å | 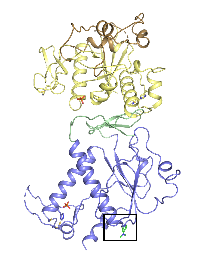  MTase Other |
| 5SL9 |   Z54571979 | 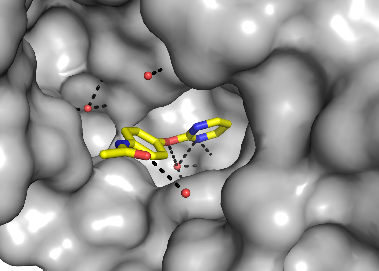 |   1.75 Å | 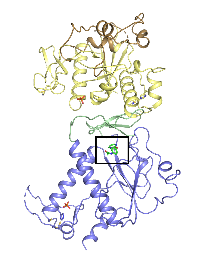  MTase Active |
| 5SLA |   Z1003207278 | 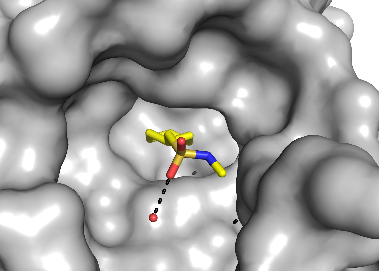  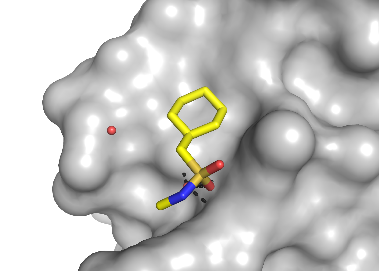 |     1.70 Å | 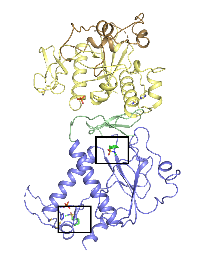  MTase Active & MTase Other |
| 5SLB |   Z744930860 | 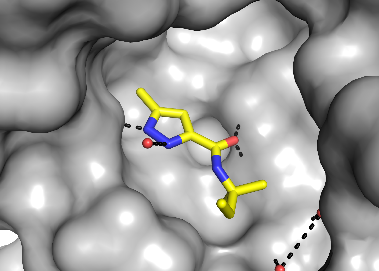 |   1.80 Å | 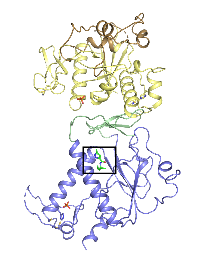  MTase Active |
| 5SLC |   Z1849009686 | 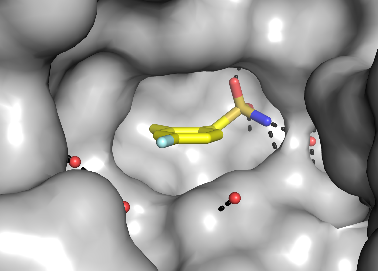 |   1.67 Å | 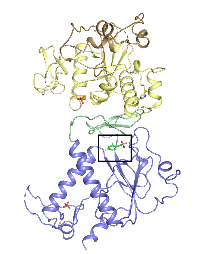  MTase Active |
| 5SLD |   Z1246465616 | 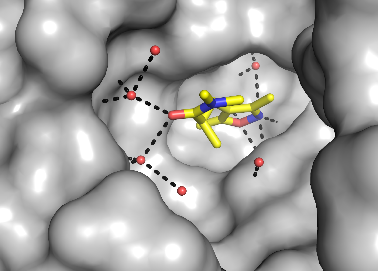 |   1.58 Å | 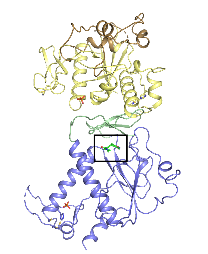  MTase Active |
| 5SLE |   Z56880342 | 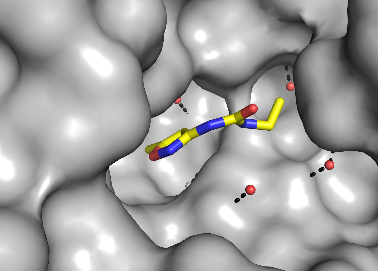  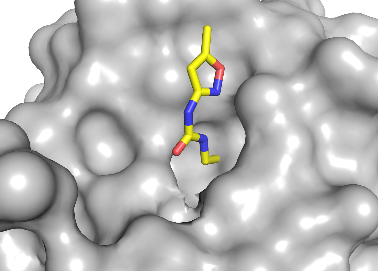 |     2.01 Å | 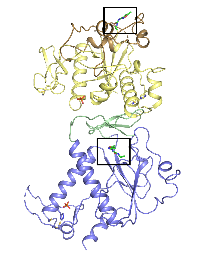  MTase Active & NSP10 Interface |
| 5SLF |   Z198195770 | 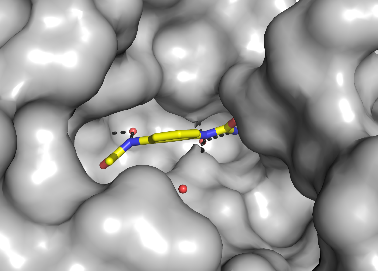  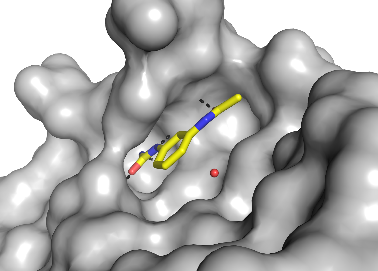 | 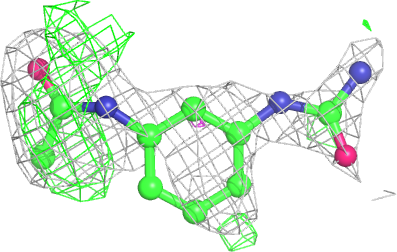    2.01 Å | 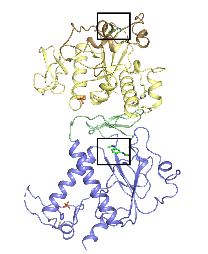  MTase Active & NSP10 Interface |
| 5SLG |   Z32400357 | 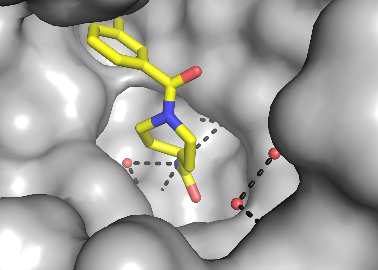 |   1.97 Å | 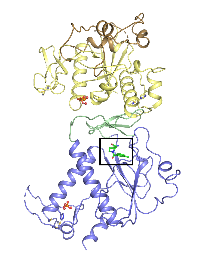  MTase Active |
| 5SLH |   Z65532537 | 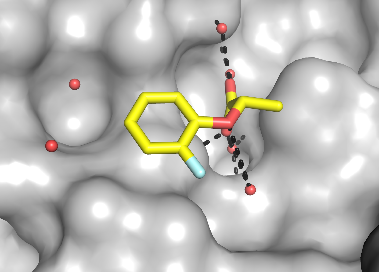 |   1.82 Å | 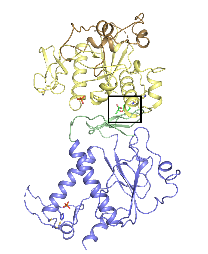  Hinge Pocket 1 |
| 5SLI |   Z1003146540 | 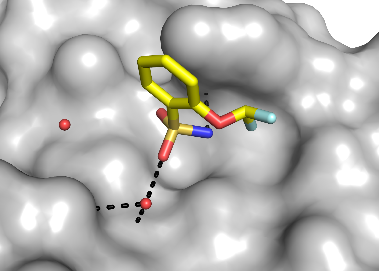  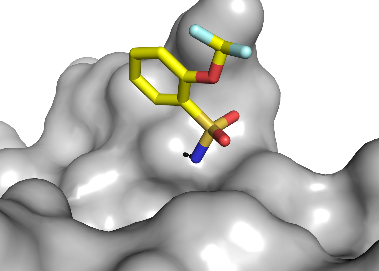  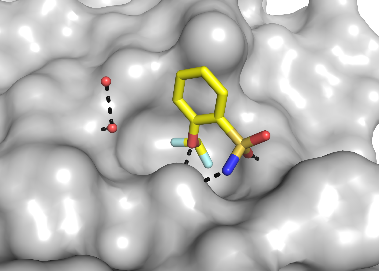 |       1.99 Å | 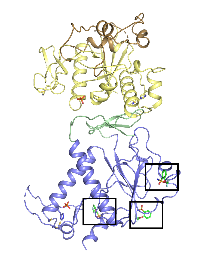  MTase Allosteric & MTase Other & MTase Other |
| 5SLJ |   Z1430613393 | 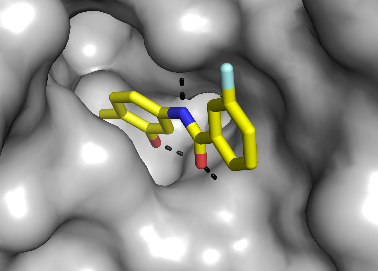 |   1.90 Å | 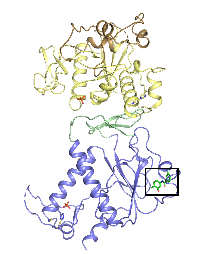  MTase Allosteric |
| 5SLK |   Z1354370680 | 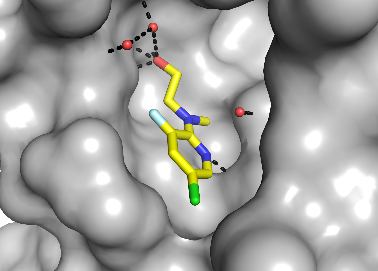 |   2.21 Å | 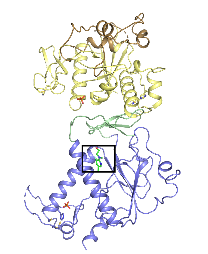  MTase Active |
| 5SLL |   Z54615640 | 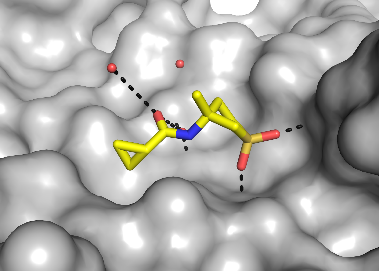 |   1.81 Å | 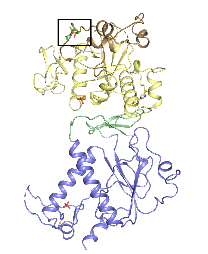  NSP10 Interface |
| 5SLM |   Z28290384 | 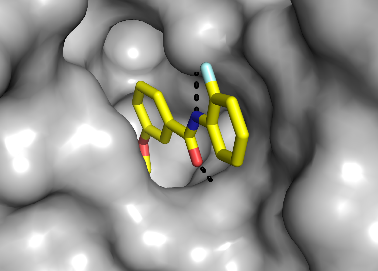 | 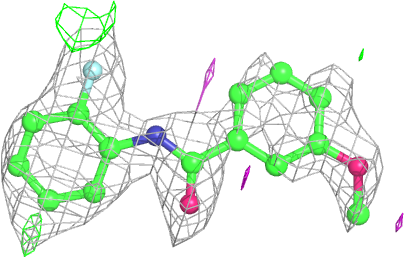  2.05 Å | 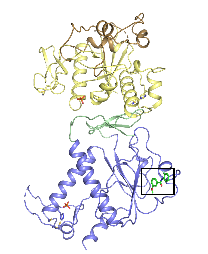  MTase Allosteric |
| 5SLN |   Z57299529 | 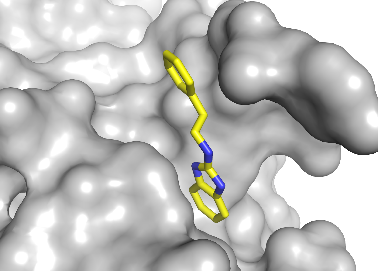 |   2.21 Å | 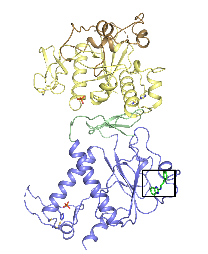  MTase Allosteric |
| 5SLO |   Z56983806 | 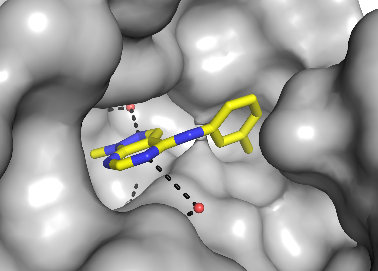 |   1.83 Å | 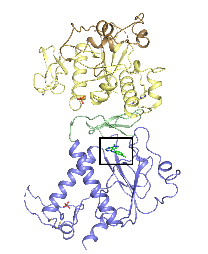  MTase Active |
| 5SLP |   Z373768898 | 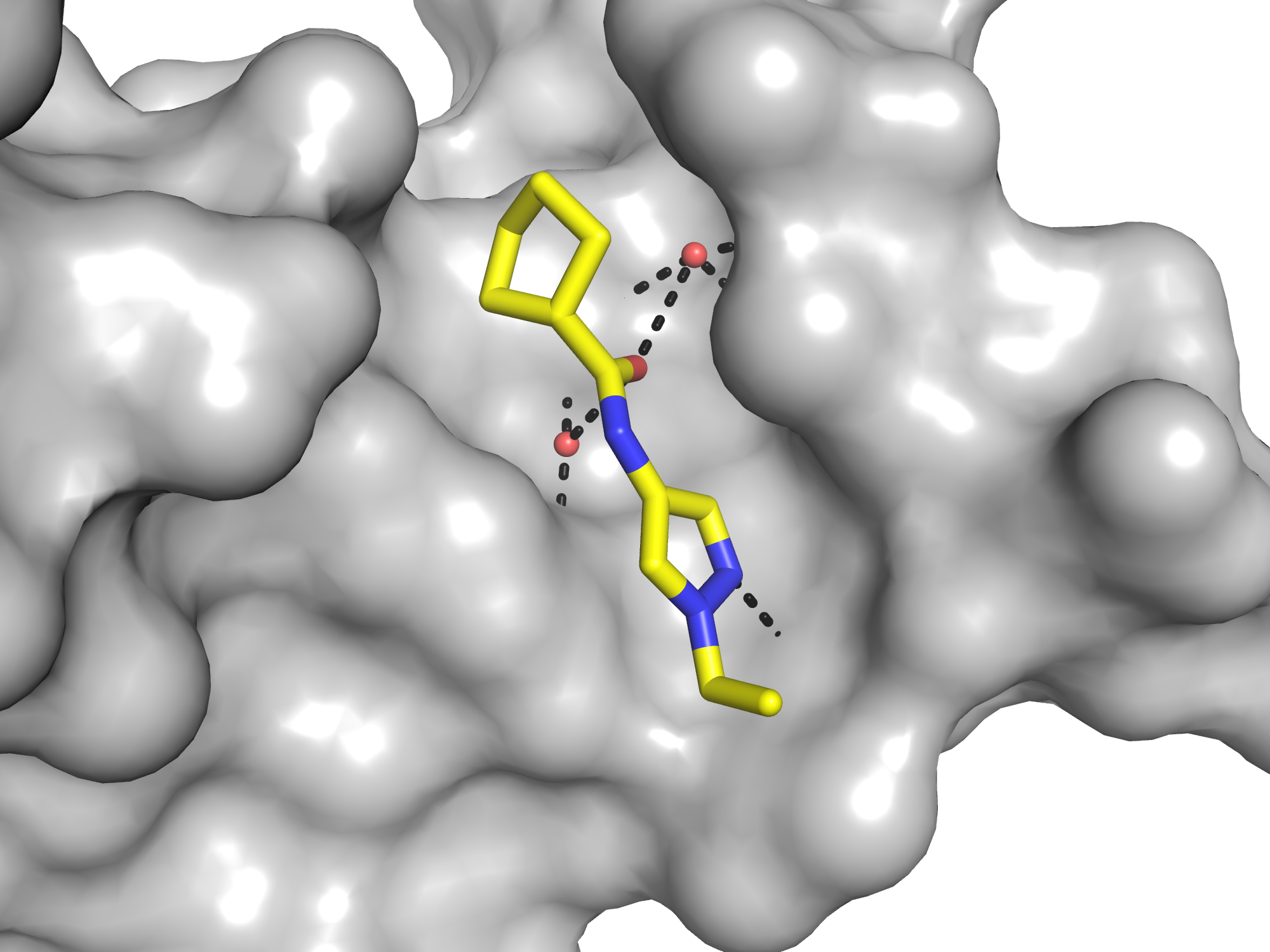 |   1.82 Å | 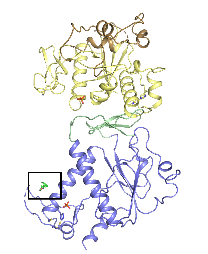  MTase Other |
| 5SLQ |   Z2856434829 | 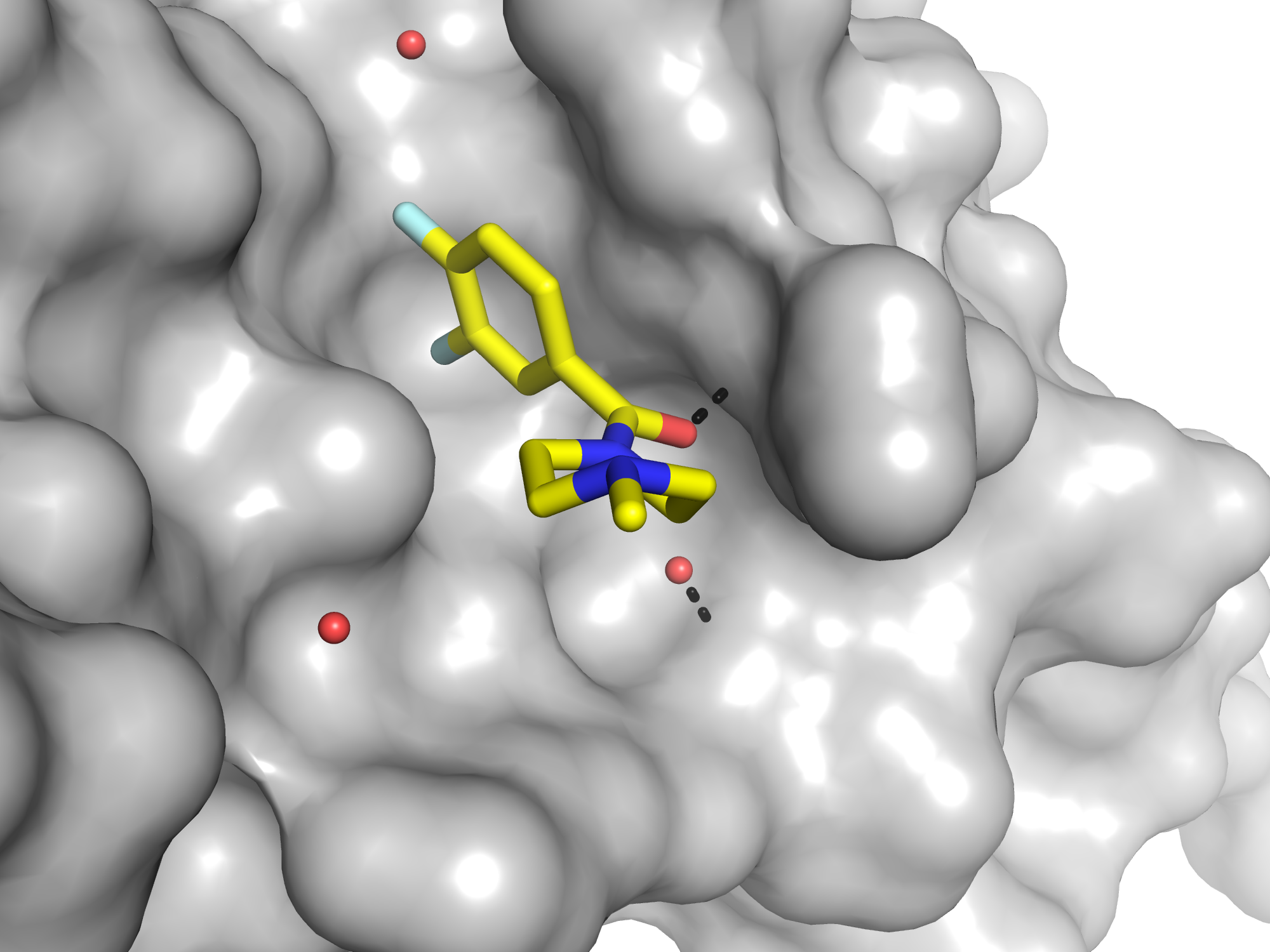 | 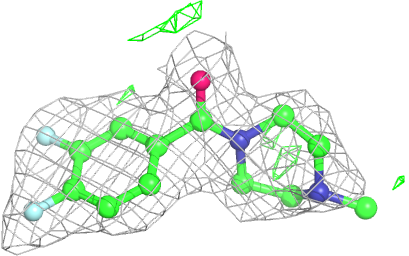  2.11 Å | 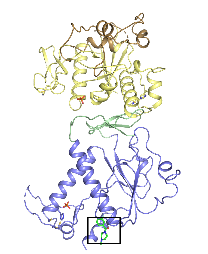  MTase Other |
| 5SLR |   Z2073741691 | 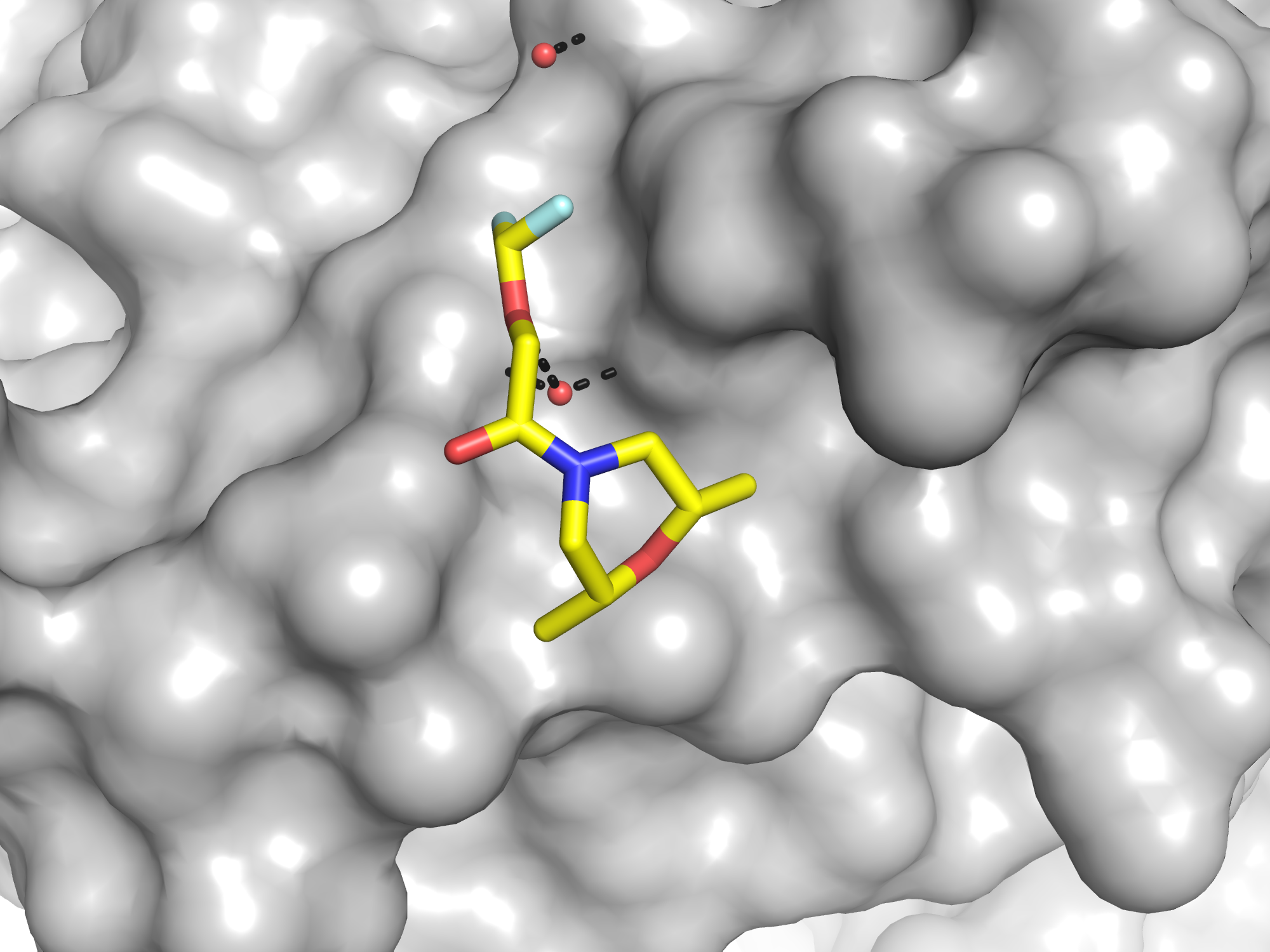 |   1.86 Å | 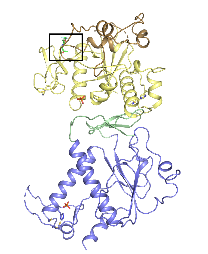  NSP10 Interface |
| 5SLS |   Z1373445602 | 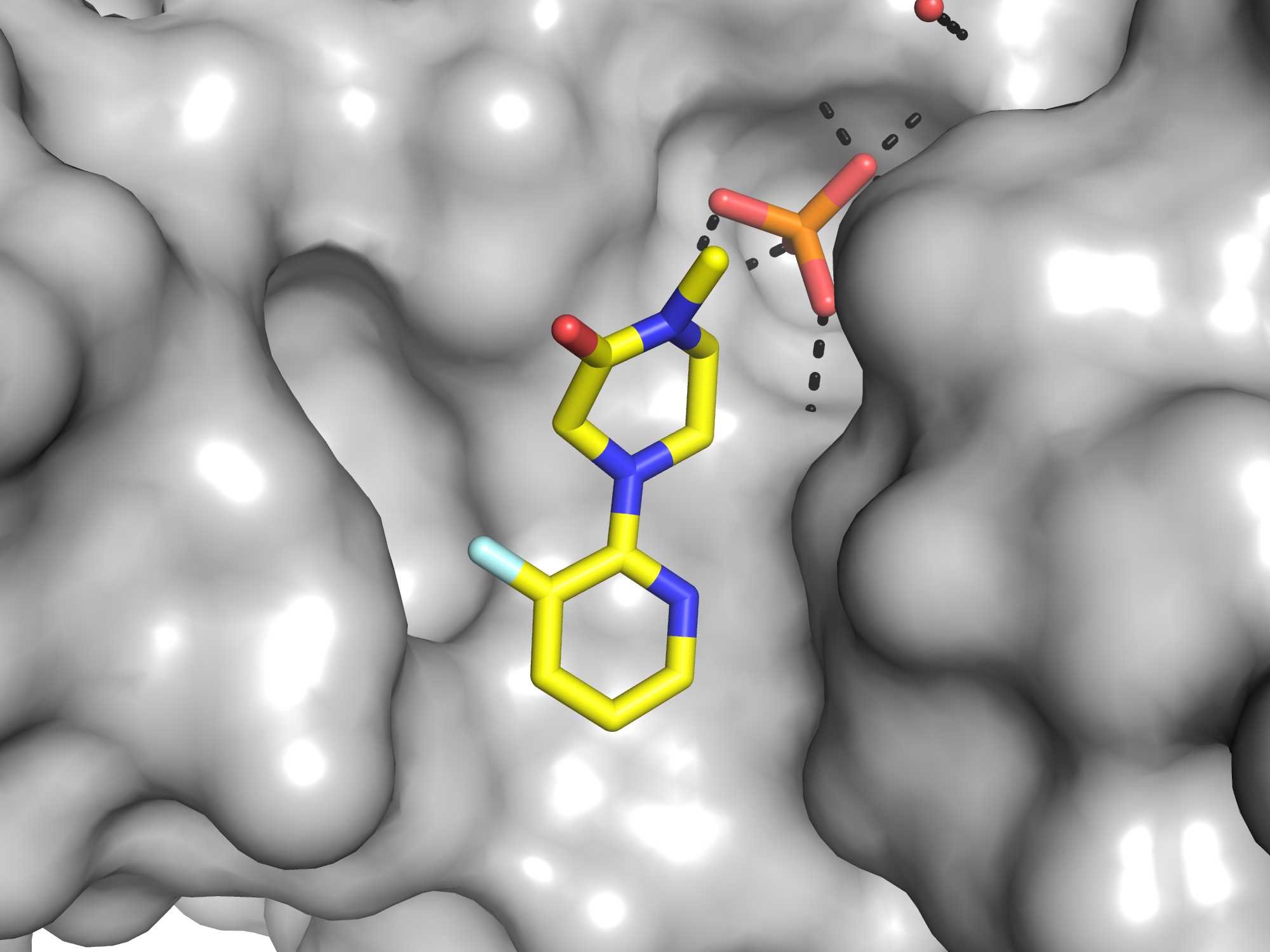 |   2.29 Å | 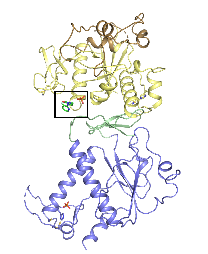  Hinge Pocket 2 |
| 5SLT |   Z1816233707 | 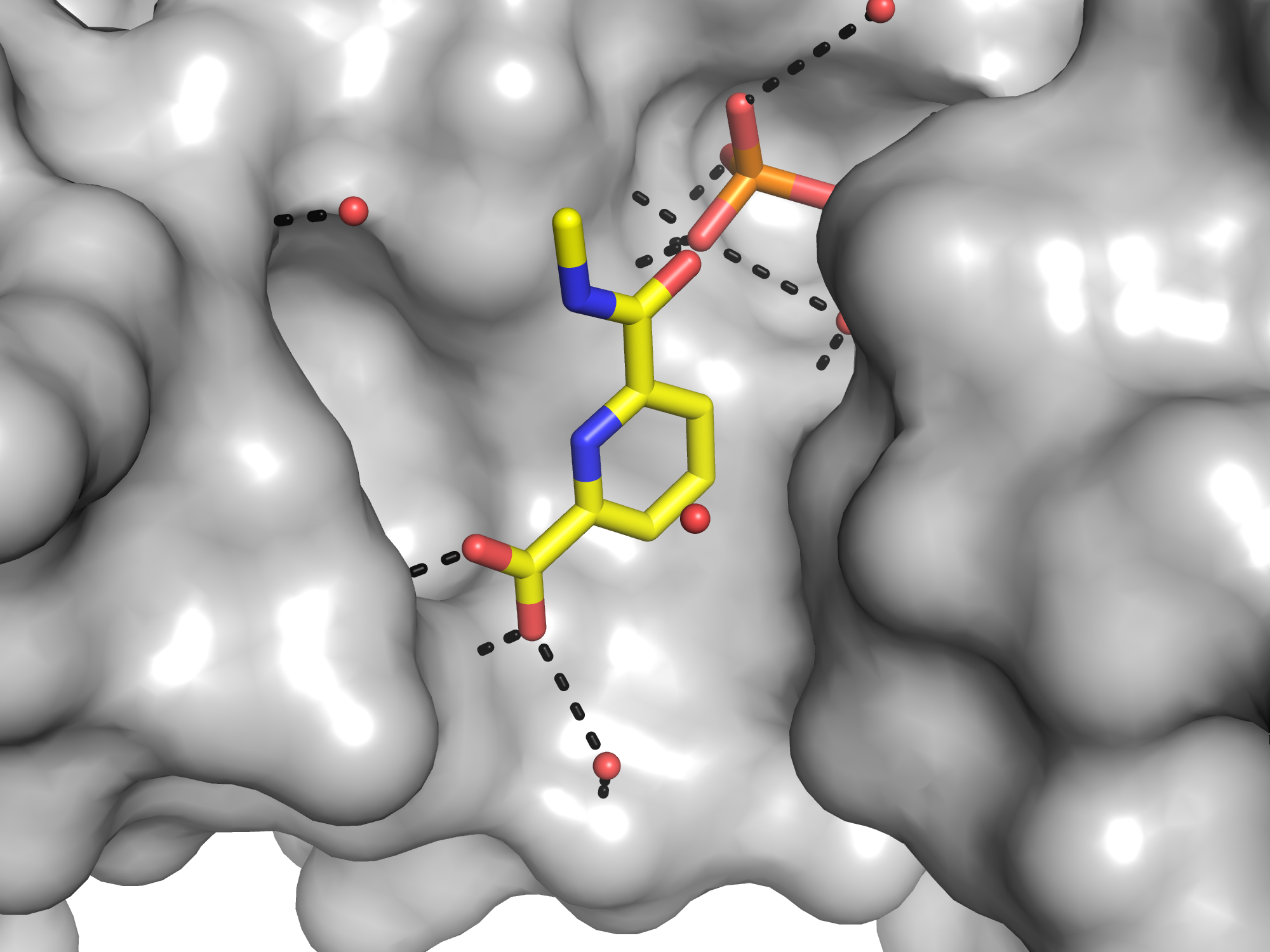 |   1.90 Å | 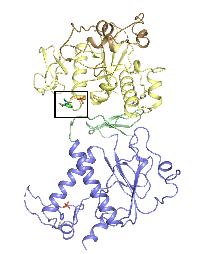  Hinge Pocket 2 |
| 5SLU |   Z1796014543 | 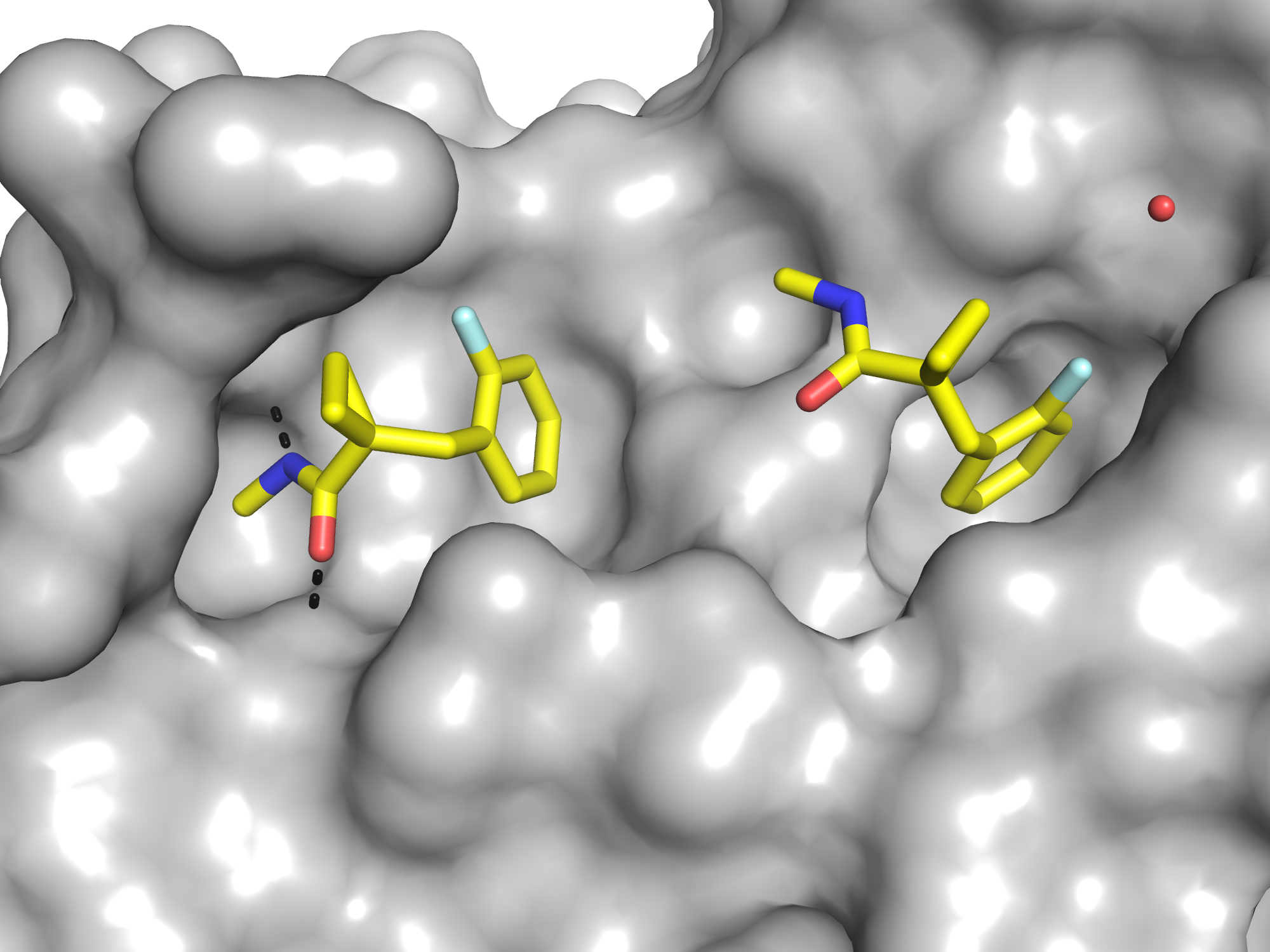 |     2.09 Å | 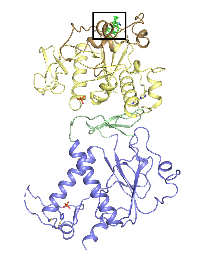  NSP10 Interface &  NSP10 Interface |
| 5SLV |   Z2856434942 | 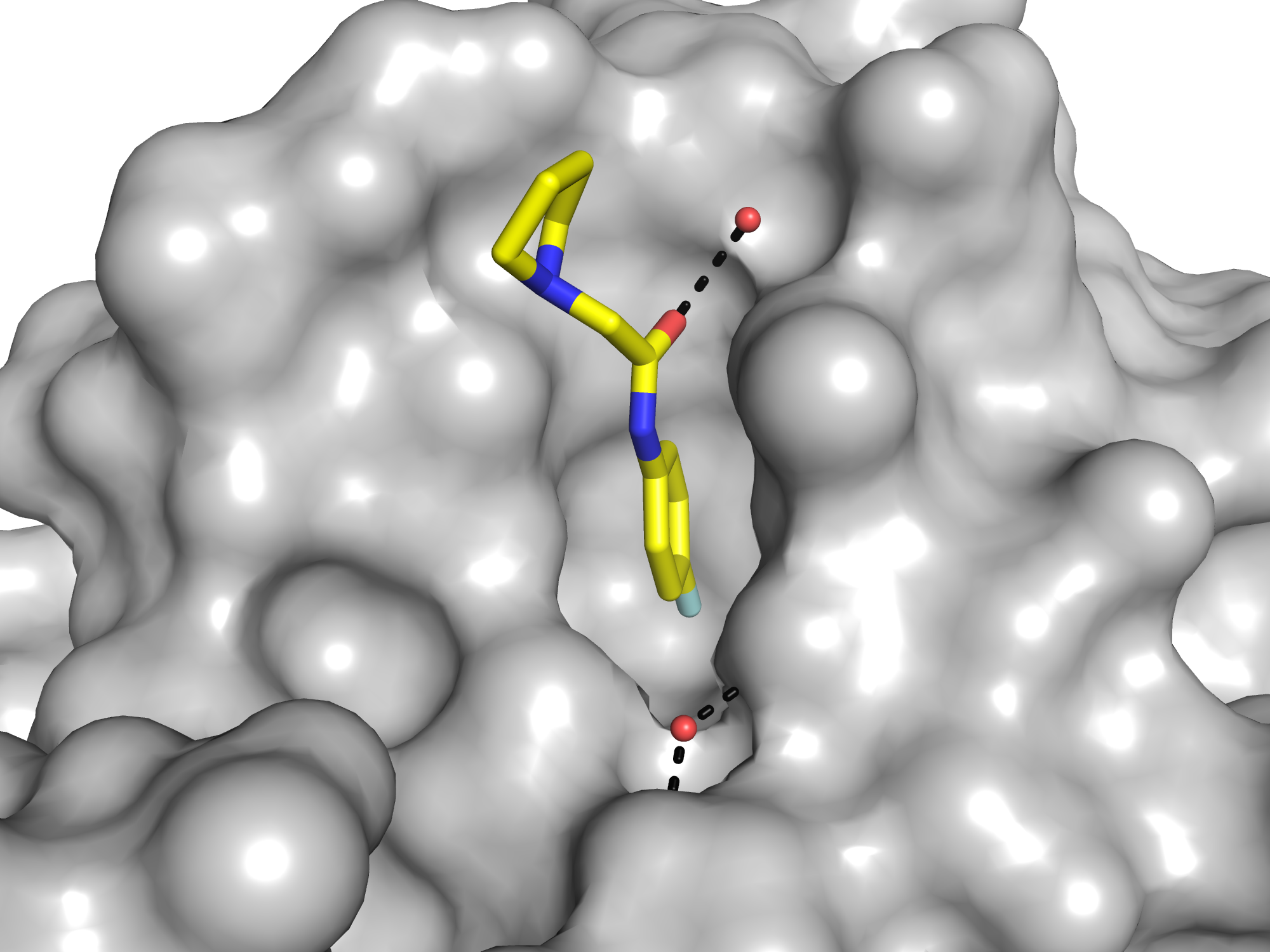 | 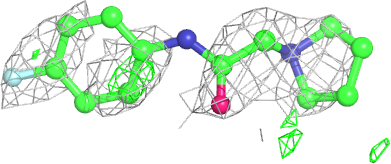  2.05 Å | 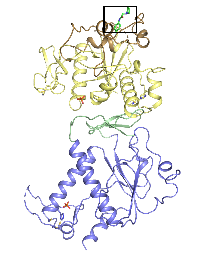  NSP10 Interface |
| 5SLW |   Z1310876699 | 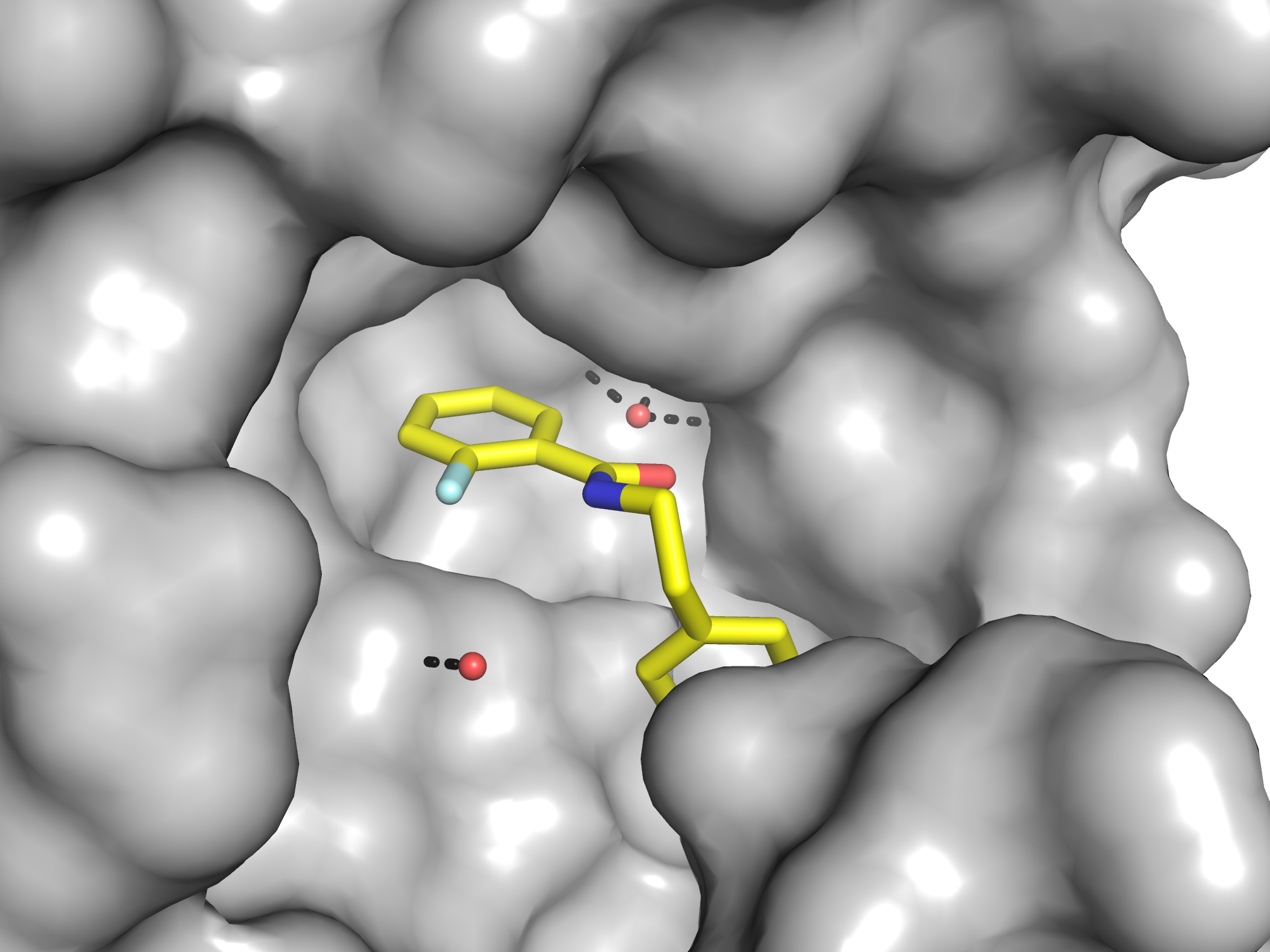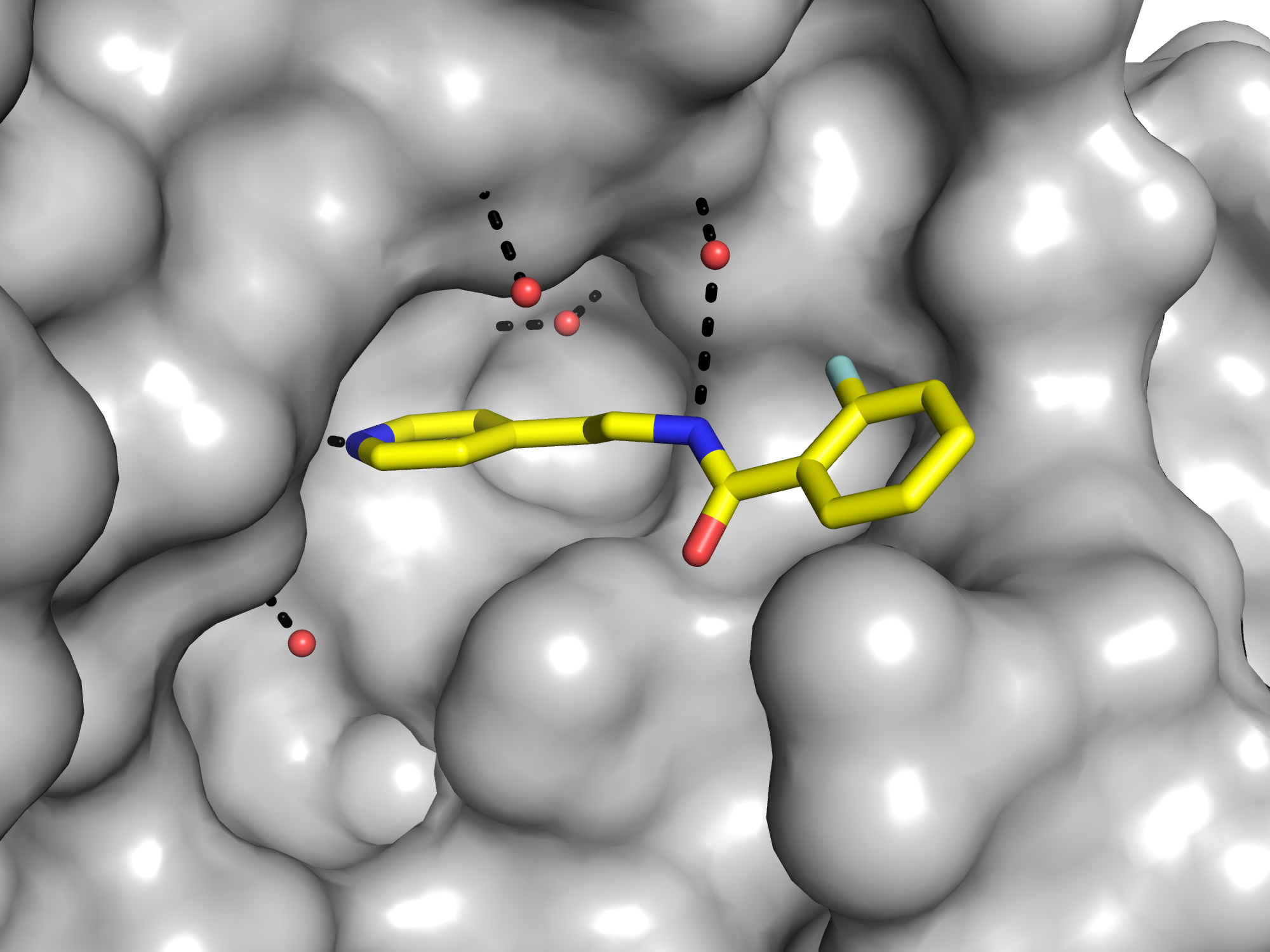 |     2.05 Å | 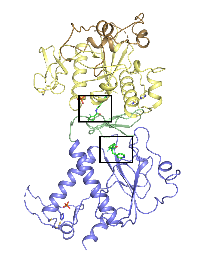  MTase Active & Hinge Pocket 1 |
| 5SLX |   Z752989138 | 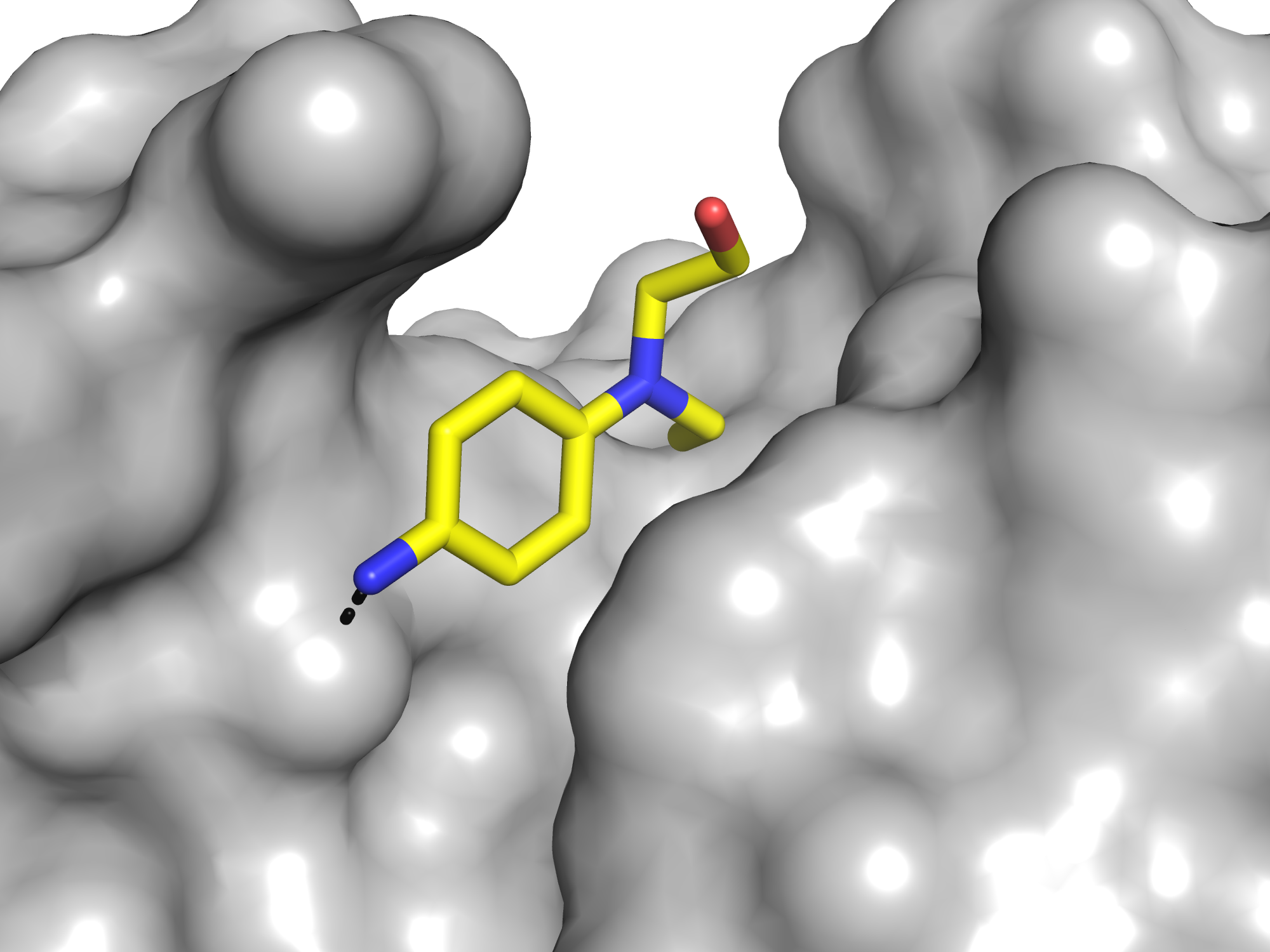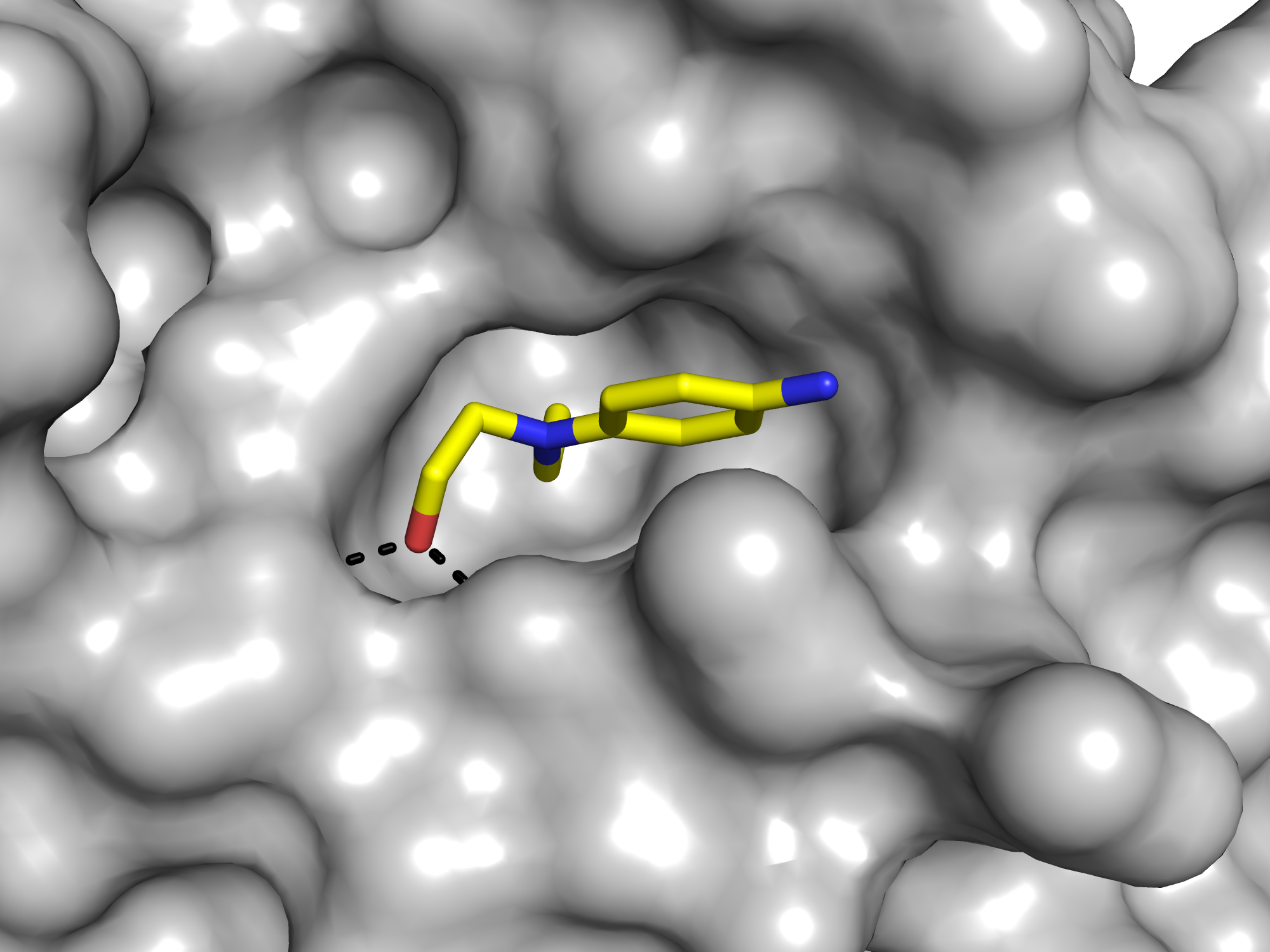 |     1.76 Å | 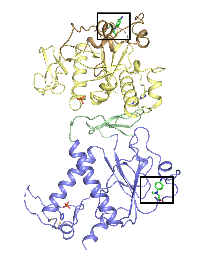  NSP10 Interface & MTase Allosteric |
| 5SLY |   Z1526504764 | 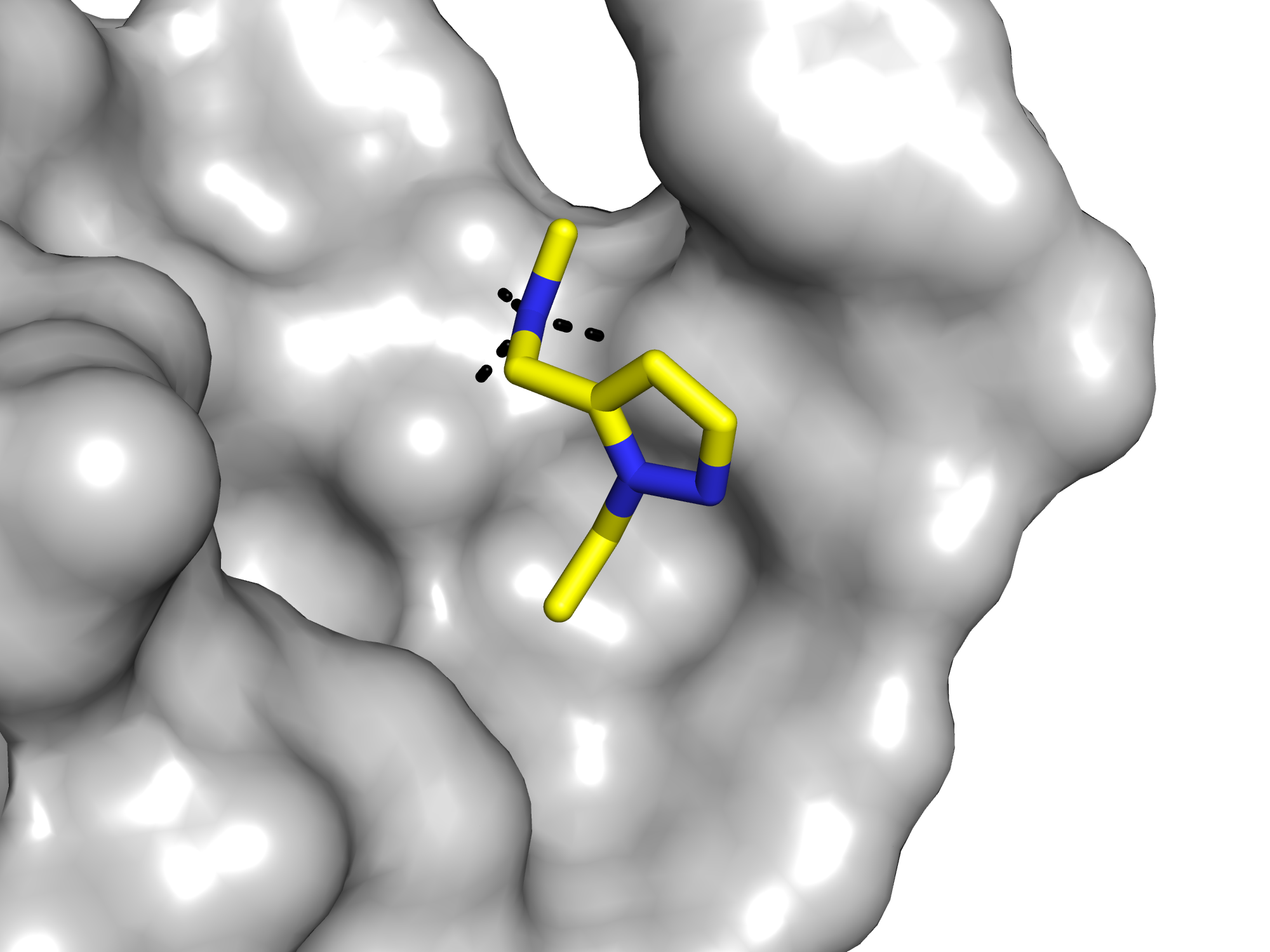 |   2.02 Å | 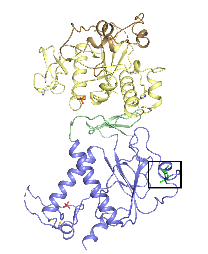  MTase Allosteric |
| 5SLZ |   Z2072621991 | 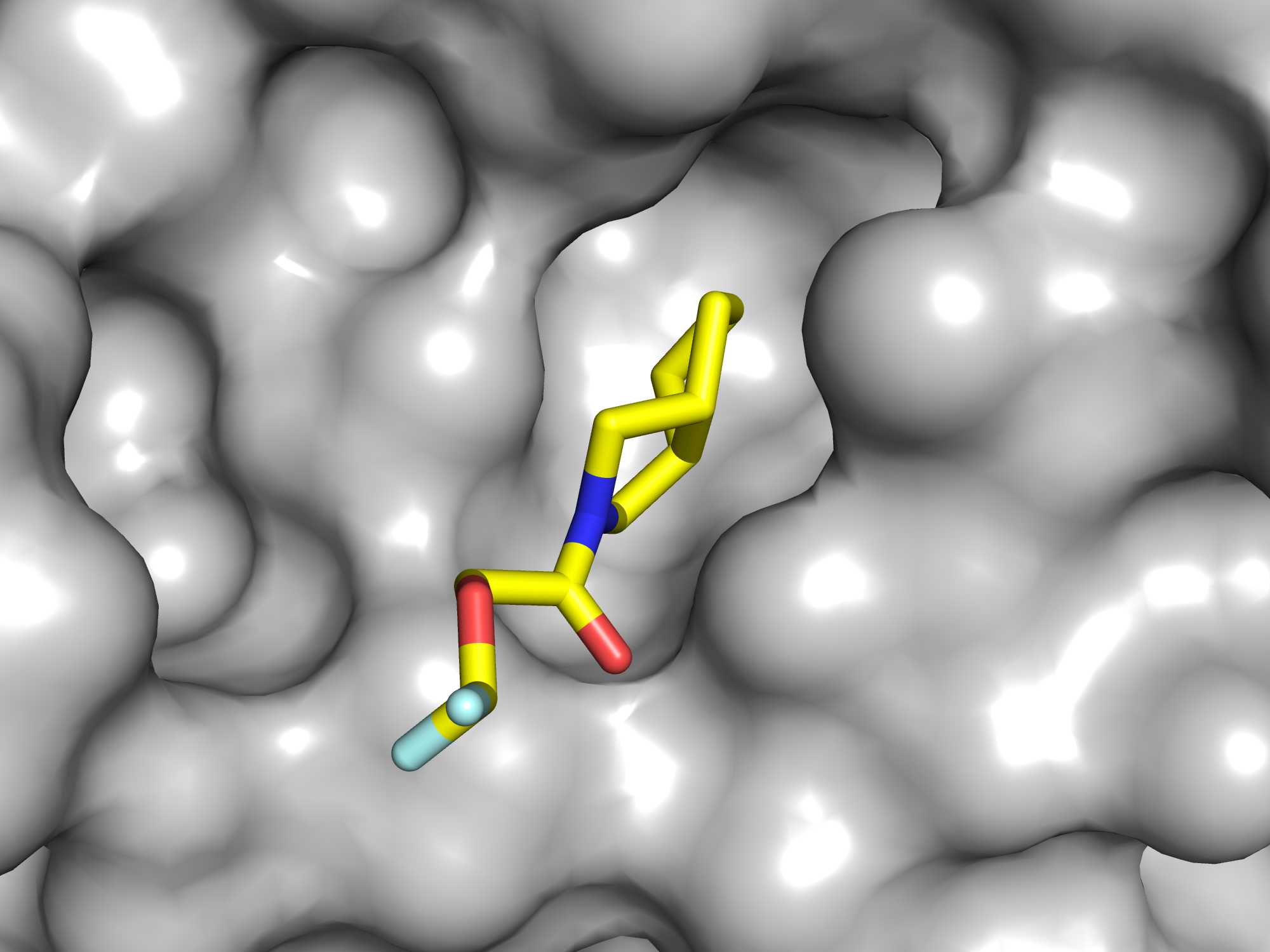 |   2.54 Å | 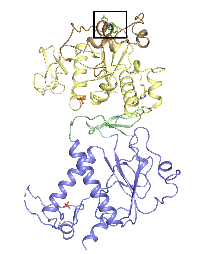  NSP10 Interface |
| 5SM0 | Z32665176 |  | 2.09 Å | MTase Active |
| 5SM1 | Z68277692 |  | 1.94 Å | MTase Other |
| 5SM2 | Z3006151474 |  | 1.78 Å | MTase Active & NSP10 Interface |
| 5SM3 | Z943693514 |  | 2.20 Å | MTase Active |
| 5SM4 | Z2856434944 |  | 2.16 Å | MTase Allosteric |
| 5SM5 | Z2856434807 |  | 1.95 Å | MTase Other |
| 5SM6 | Z1899842917 |  | 2.29 Å | Hinge Pocket 1 |
| 5SM7 | Z1247413608 |  | 1.95 Å | MTase Active |
| 5SM8 | Z2027158783 |  | 1.95 Å | MTase Active |
| 5SM9 | Z2234920345 |  | 2.01 Å | MTase Allosteric |
| 5SMA | Z2856434890 |  | 2.01 Å | MTase Allosteric |
| 5SMB | Z419995480 |  | 2.18 Å | MTase Active |
| 5SMC | Z2033637875 |  | 2.19 Å | MTase Active & MTase Allosteric |
| 5SMD | Z274575916 |  | 1.83 Å | Exon Active |
| 5SME | Z437584380 |  | 1.91 Å | NSP10 Interface |
| 5SMF | Z56791867 |  | 2.01 Å | NSP10 Interface & NSP10 Interface |
| 5SMG | Z2092370954 |  | 1.87 Å | MTase Active |
| 5SMH | Z2856434938 |  | 2.64 Å | MTase Active |
| 5SMI | Z71580604 |  | 2.08 Å | NSP10 Interface |

**Supplementary Table 2 –** Details of all the fragment structures included in this study showing PDB codes, compound structure and codes, pocket details with polar contacts shown in black dashes, fragment locations and electron density maps. Maps shown are 2F_o_-1F_c_ at 0.7 σ in grey with F_o_-F_c_ difference maps contoured at +3 in green and -3 in purple with map resolution indicated.
